# Electrochemical sensor based on Ni-exchanged natural zeolite/carbon black hybrid nanocomposite for determination of vitamin B_6_

**DOI:** 10.1007/s00604-021-04992-x

**Published:** 2021-09-06

**Authors:** Radosław Porada, Katarzyna Fendrych, Bogusław Baś

**Affiliations:** Department of Analytical Chemistry and Biochemistry, Faculty of Materials Science and Ceramics, AGH University of Science and Technology, Mickiewicza 30, 30-059 Krakow, Poland

**Keywords:** Hybride nanocomposite, Natural zeolite, Carbon black, Vitamin B_6_, Voltammetry, Modified electrode, Dietary supplements, Electrochemical sensor

## Abstract

The utilization of environmentally friendly nanoporous natural zeolite exchanged with Ni^2+^ ions (NiZ) and conductive carbon black (CB) in the fabrication of a novel and selective voltammetric sensor of vitamin B_6_ (VB_6_) is presented. The used clinoptilolite-rich zeolite material and CB were characterized in terms of morphology and textural properties. The superior properties of Ni-zeolite/carbon black modified glassy carbon electrode (NiZCB-GCE), arising from the synergistic effect of combining the unique features of zeolite and conductive carbon black, were confirmed by cyclic voltammetry (CV) and electrochemical impedance spectroscopy (EIS) measurements. In the determination of VB_6_ with the use of differential pulse voltammetry (DPV), the optimization of the pH value of supporting electrolyte and instrumental parameters, as well as the interference study were performed. Under optimized conditions, the oxidation peak current at the potential +0.72 V vs. Ag | AgCl | 3 M KCl reference electrode was linear to the VB_6_ concentration in the range 0.050 to 1.0 mg L^−1^ (0.30–5.9 μmol L^−1^) (*R* = 0.9993). The calculated limit of detection (LOD, *S*/*N* = 3), equal to 15 μg L^−1^ (0.09 μmol L^−1^), was much better compared to chemically modified electrodes with other carbon-based materials. The RSD for 0.5 mg L^−1^ was in the range 2.5–5.4% (*n* = 4). The developed NiZCB-GCE was successfully applied to the determination of VB_6_ in commercially available multivitamin dietary supplements, food, and water samples. The obtained recoveries ranged from 95 to 106%.

**Supplementary Information:**

The online version contains supplementary material available at 10.1007/s00604-021-04992-x.

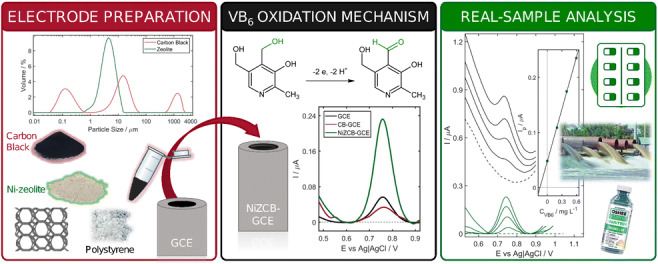

## Introduction

Vitamin B_6_ (VB_6_) is a part of water-soluble B complex vitamins and is found in chemically related forms, i.e., pyridoxine (PN), pyridoxal (PL), pyridoxamine (PM), and their phosphorylated derivatives [1]. The most stable form is PN, which constitutes a component of multivitamin and vitamin B_6_ supplements and enriched food. Pyridoxine is metabolized inside of the human body into the biologically active form, i.e., pyridoxal 5′-phosphate (PLP), which acts as a cofactor for a large number of enzymatic reactions in amino acid, glucose, and lipid metabolism [2], as well as in neurotransmitter synthesis, hemoglobin formation and functioning, and gene expression [3]. VB_6_ has a role in improving immune functioning [1], as its anti-inflammatory activity reduces the risk of viral infection [4]. The latest studies show a potential role of vitamin B_6_ in ameliorating the severity of COVID-19 and its complications [5].

In view of the above, the dietary supplementation of vitamin B_6_ has gained more attention over the last years, and in consequence large consumption of drug formulation such as multivitamins, vitamin B complex, and vitamin B_6_ supplements is observed. It in turn involves the necessity to develop simple and accurate analytical methods for VB_6_ quantitative analysis in pharmaceuticals in order to ensure their high quality. Up to now, i.e., chromatographic [6], spectrophotometric [7], potentiometric [8], electrophoretic [9], chemiluminescence [10], and voltammetric [11] approaches were applied for vitamin B_6_ determination. For VB_6_ voltammetric detection, different kinds of working electrodes, such as glassy carbon (GC) [12], carbon paste (CP) [13], boron-doped diamond (BDD) [14], and mercury [15] electrode, were initially reported. In order to improve the analytical performance of a VB_6_ electrochemical sensor, more and more functional materials have been used to fabricate chemically modified electrodes (CMEs). These include the application of metal nanoparticles, like gold [16] and copper [17], as well as metal oxide nanoparticles, such as Au-CoO core-shell [18] or ZrO_2_ [19]. Recently, the implementation of carbon-based materials, like single-walled carbon nanotube (SWCNT) and multi-walled carbon nanotubes (MWCNT) [20], electrochemically reduced graphene oxide (ERGO) [21], and ordered mesoporous carbon (OMC) [22], has also been reported. Nevertheless, the utilization of the above-mentioned nanomaterials in electrode modification can be limited by some of their drawbacks, such as metal oxides nanoparticle instability, leading to the deterioration of their quality, high chemical reactivity, biological harmfulness, explosive character, and difficulties in their synthesis, related to the employment of hazardous and expensive chemicals, high energy input with a negative effect on the environment [23]. Similarly, carbon-based materials possess some limitations in their application in sensor fabrication due to their structural characteristics and properties, such as chemical activation, surface functionalization, defects, and particle aggregation [24].

In order to overcome these limitations, the explorations of new sensing materials are reasonable. From that point of view, one of the interesting electrode modifiers are zeolites, which constitute a wide group of micro- and mesoporous, natural or synthetic, crystalline, hydrated aluminosilicates with the specific framework structure. Zeolites indicate unique molecular sieving properties, offering both shape and size discrimination/recognition, as well as high cation exchange capacity (CEC). The ability to undergo the ion-exchange process with various electroactive species leads to catalytical reactivity of zeolites, which is exploited in the development of the novel electrochemical sensor of different organic, inorganic, and pharmaceutical compounds [25–27]. Since zeolites possess a charge selective feature, they can be used to determine analytes in the presence of potential interferences presented in physiological conditions, which is crucial in all areas of medicinal chemistry [28]. The above-mentioned advantages coupled with the possibility to apply zeolites as an effective sorbent of vitamins [29], as well as their excellent stability in chemical and thermal processes, the availability in nature, and low cost of exploration, make zeolites an attractive sensing material in comparison with the ones reported previously.

In recent decades, the utilization of zeolites in the design of electrochemical sensors has led to the formation of a subcategory of CMEs, called zeolite-modified electrodes (ZMEs), which are extensively studied in respect of the methods of their preparation, the charge-transfer mechanism, and analytical applications [30, 31]. Because zeolites are electronic insulators and possess crystalline powder character, many different methods have been applied for their confinement at the electrode surface. Two of the most common approaches appearing in the literature are related to the dispersion of zeolite microparticles within conducting carbon-based composite matrices or deposition of a zeolite-polymeric film (with or without carbon) on the solid-electrodes surface [32]. In both strategies, the application of graphite as an electron-conducting component of ZMEs has been reported so far [33, 34]. To the best of our knowledge, the utilization of other carbon-based nanomaterials, such as carbon black (CB), has never been presented. CB provides many advantages in the electrodes surface modification, such as large specific surface area, low density, disordered structure, electrochemical stability, and high electron conductivity, which in association with features of electrochemically attractive materials, like zeolites, can lead to significant improvement of the analytical performance of sensors [35].

In this work, the combination of the extremely valuable properties of zeolites and conductive carbon black Printex XE2-B in the fabrication of a novel voltammetric sensor of vitamin B6 was presented for the first time. As far as we know, the application of Ni-exchanged form of environmentally friendly nanoporous natural zeolite, as well as the replacement of extensively used graphite as an electron-conducting component of carbon-based ZMEs by conductive CB, has never been reported. Zeolite and CB were analyzed with regard to the particle size distribution, specific surface area, porosity, and morphology. The Ni-zeolite/carbon black modified glassy carbon electrode (NiZCB-GCE) was fabricated by applying Ni-zeolite-carbon black polystyrene layer on the surface of the glassy carbon electrode (GCE). With the aim to specify the advantages of CB and NiZ utilization in the proposed sensor, the NiZCB-GCE was characterized electrochemically by means of cyclic voltammetry (CV) and electrochemical impedance spectroscopy (EIS) and compared with the behavior of the bare GCE, as well as GCE modified with Ni-zeolite (NiZ-GCE), carbon black (CB-GCE), Ni-zeolite/graphite (NiZG-GCE), and Mn-zeolite/carbon black (MnZCB-GCE) layer. Finally, the analytical usefulness of the proposed sensor was confirmed by its successful application in the voltammetric determination of VB_6_ in commercially available multivitamin dietary supplements, energy drink, and spiked water samples. In order to verify the obtained results, UV-Vis spectroscopy was applied as a reference method.

## Experimental

### Chemicals and reagents

The Ukrainian-Transcarpathian zeolite material of volcanic-sedimentary origin, containing 65.1% of Ca-clinoptilolite was received from the Andalusia Group (Poznań, Poland). Conductive carbon black Printex® XE2-B in the form of granules was purchased from Evonik Degussa GmbH, Inorganic Materials (Frankfurt, Germany) and used without any pretreatment. Tetrahydrofuran and dichloromethane, glucose and cetrimonium bromide (CTAB) were obtained from Avantor Performance Materials Poland S.A. Graphite powder (<20 μm, synthetic), polystyrene (average *M*_w_ ~ 35,000), caffeine, Triton X-100, sodium dodecyl sulfate (SDS), humic acid (HA), titanium dioxide, starch, and magnesium stearate, as well as vitamin B_1_ (thiamine hydrochloride, reagent grade, ≥99%), vitamin B_3_ (niacin, USP reference standard), vitamin B_5_ (D-pantothenic acid hemicalcium salt, ≥98%), and vitamin B_12_ (cyanocobalamin, ≥98%), were purchased from Sigma-Aldrich. The standard stock solution of vitamin B_6_ in the form of pyridoxine (≥98%, Sigma-Aldrich) at the concentration of 1000 mg L^−1^ was prepared weekly, protected from the light, and stored at 4 °C in the fridge. A working solution of VB_6_ at the concentration of 100 mg L^−1^ was prepared daily through dilution of the standard VB_6_ stock solution with distilled water. Phosphate buffer solution with pH from 6 to 8 and acetate buffer solution with pH between 4 and 6 were obtained by mixing appropriate volume of 0.2 mol L^−1^ NaH_2_PO_4_ with 0.2 mol L^−1^ Na_2_HPO_4_, and 1.0 mol L^−1^ CH_3_COOH with 1.0 mol L^−1^ CH_3_COONa, respectively (Avantor Performance Materials Poland S.A.). All applied chemical reagents were of analytical grade and used as received. For the preparation of all aqueous solutions, double-distilled water was used.

### Apparatus and software

The X-ray powder diffraction X’Pert system (CuKα) (Philips, the Netherlands) was used with the aim to establish the phase composition of the zeolite material. The collected XRD pattern, in the 2*ϴ* angle range of 5–70° with a step of 0.016°, was interpreted based on X’Pert HighScore Plus software and the International Centre for Diffraction Data (ICDD). The quantitative phase composition was determined using the Rietveld method.

Conductive carbon black and zeolite were characterized in terms of their particle size distribution, specific surface area, porosity, and morphology. In order to establish particle size distribution (PSD), the dynamic light scattering (DLS) method was used. The DLS measurements were carried out using Zetasizer Nano-ZS (Malvern Instruments, UK) with dedicated software Mastersizer 2000 ver. 5.60. The specific surface area (SSA) and porosity of zeolite powder and carbon black were set out using the multipoint nitrogen adsorption method at 77 K (ASAP 2010, Micrometric Instruments, USA). Before measurement, the samples were degassed at 378 K for 24 h. The Brunauer-Emmett-Teller (BET) and the Barret-Joyner-Halenda (BJH) methods were used in order to determine SSA and porosity, respectively. With the use of the FEI Nova NanoSEM 200 scanning electron microscope (ThermoFisher Scientific, USA), the morphology of zeolite, carbon black, and modifying layer was studied. Energy-dispersive X-ray spectroscopy EDS attachment was used for elemental analysis of the initial zeolite material, as well as the zeolite after modification with Ni^2+^ and Mn^2+^ cations. Prior to measurements in a vacuum, the samples were sprayed with graphite.

All voltammetric measurements were performed using the M164 electrode stand connected to the M161 multipurpose electrochemical analyzer (both mtm-anko, Poland) with the EALab 2.1 software. The standard three-electrode quartz cell with a volume of 10 mL, composed of platinum wire auxiliary electrode, double-junction silver-silver chloride reference electrode (Ag | AgCl | 3 M KCl), and the bare or modified GCE (MF-2012, *φ* = 3 mm, BASi, USA) as a working electrode was applied in the entire experimental work. In the electrochemical impedance spectroscopy (EIS) measurements, μAUTOLAB III analyzer (EcoChemie, the Netherlands) with NOVA 2.0 software was used. The homogeneity of the solution was ensured by stirring at approximately 200 rpm with the use of a magnetic bar coated with Teflon®. SevenCompact S210 laboratory pH-meter (Mettler Toledo, Switzerland) was utilized to adjusted the pH value of buffer solutions.

The double-beam JASCO V-630 UV-VIS spectrophotometer (JASCO Deutschland GmbH, Germany) fitted with deuterium and halogen lamps was applied in the spectrophotometric measurements, which were performed using a 10-mm quartz cuvette and double-distilled water as a reference sample. The spectra were collected in the range of 200 to 500 nm with the speed of spectrum registration of 200 nm min^−1^ and 2.0 nm slit width.

### Procedures

#### Zeolite modification and electrode fabrication

Natural zeolite material with high content of Ca-clinoptilolite was transformed into sodium form by mixing with 2 mol L^−1^ aqueous solution of NaCl (4 mL of the solution per 1 g of zeolite) and shaking for 2 h. After that, the suspension was left for 24 h at 25 °C to ensure the contact of the zeolite with the solution. Thereafter, zeolite was centrifuged, rinsed up several times with double-distilled water (AgNO_3_ test), dried, and homogenized. The Na^+^-ions of the zeolite were exchanged with Ni^2+^ and Mn^2+^ ions (separately), which were introduced into the zeolite structure from 0.1 mol L^−1^ aqueous solution of NiCl_2_·6H_2_O and MnCl_2_·4H_2_O, receptively. For this purpose, 0.5 g of zeolite was contacted two times with 5 mL of the appropriate metal salt solution, shaken for 24 h at 25 °C, and centrifuged at 15000 rpm for 10 min. Afterward, the samples were washed repeatedly with a small volume of double-distilled water and dried at room temperature.

The obtained form of Ni-zeolite was subsequently used in the preparation of two kinds of modified electrodes, where CB or graphite (G) was applied as an electron-conducting component. The fabrication of NiZCB-GCE and NiZG-GCE took places in several stages: (1) the mixture of Ni-zeolite and conductive carbon black, as well as Ni-zeolite and graphite, at a weight ratio of 1:1 was prepared by thorough grinding of the components in the agate mortar; (2) 5 mg of polystyrene was dissolved in 400 μL of tetrahydrofuran and 600 μL of dichloromethane; (3) each time, 5 mg of Ni-zeolite/carbon black and Ni-zeolite/graphite mixture were weighted, transferred to two tubes for microcentrifugation, and connected with 500 μL of the above-mentioned solvent containing polystyrene; (4) the resulting suspensions were homogenized using Ultrasonic bath IS-1 (InterSonic, Poland) for 15 min; (5) the surface of GCEs was polished with 0.3-μm alumina powder (Buehler Micropolish II, USA), cleaned in water, and rinsed several time in acetone; (6) 10 μL of each mixtures was dropped carefully onto the cleaned surface of GCE and left for 24 h in room temperature; (7) after complete solvent evaporation, the NiZCB-GCE and NiZG-GCE were ready to be used in measurements. Based on the best durability, highest stability, and favorable electrochemical parameters, the NiZCB-GCE with weigh ratio of Ni-zeolite to carbon black equal to 1:1 was chosen. In the way outlined above, MnZCB-GCE was obtained by exploiting the received form of Mn-zeolite in the modification procedure. In order to determine the effect of zeolite and CB presence on the electrochemical response of the proposed electrodes, a modifying layer containing only zeolite or CB (5 mg in each case) was prepared as described above and applied onto the GCE surface resulting in the NiZ-GCE and CB-CGE, respectively.

#### Sample preparation

Dietary supplements Vitaminum B Complex (Hasco-Lek, Poland), BallansB Complex (Madson, Poland), Bellis B Complex (Bellis Pharma Sp. z o.o., Poland), and MagB6forte (Amnivest Sp. z o.o., Poland), containing vitamin B_6_ in the form of pyridoxine (1.4 mg per tablet), as well as Vitaminum B Compositum (Teva Pharmaceuticals Sp. z o.o., Poland) and Magne B6 (Sanofi-Aventis Sp. z o.o., Poland) containing 5 mg of pyridoxine hydrochloride per tablet, were purchased at a local pharmacy. With the aim of quantitative analysis of VB_6_ content in the proposed dietary supplements, five tablets of each brand were weighed and ground in an agate mortar. Successively, the solutions of VB_6_ at the concentration of 15.0 mg L^−1^ were prepared by dissolving a proper weight amount of each tablet in 25 mL of double-distilled water and ultrasonic homogenization. The received solutions were clear and no filtration was required.

The sample of commercially available energy drink-Oshee Vitamin Mg + B_6_-was pre-treated just before measurement by filtrating with the use of syringe filter with Mixed Cellulose Ester (MCE) membrane filter with the pore diameter of 0.22 μm (Alchem Grupa Sp. z o.o, Poland).

#### Measurement procedures

To determine the range of useful potentials and investigate the influence of the cation introduced into the zeolite framework, cyclic voltammograms (CVs) for MnZCB-GCE and NiZCB-GCE were recorded. Subsequently, the impact of the electron-conductive carbon material was assessed based on the electrode performance towards 0.2 mmol L^−1^ [Fe(CN)_6_]^4−/3−^ in 0.1 mol L^−1^ KCl. The measurements were conducted by means of the cyclic voltammetry (scan rate *v* = 0.010 V s^−1^) and the electrochemical impedance spectroscopy (EIS), in which the sinusoidal signals of the frequency ranging from 100 kHz to 25 mHz and the amplitude of 10 mV (superimposed on the formal potential of the [Fe(CN)_6_]^4−/3-^ redox system) were used. Finally, the active surface area of the NiZCB-GCE was calculated based on the CVs recorded for 0.2 mmol L^−1^ [Fe(CN)_6_]^4−^ in 0.1 mol L^−1^ KCl using the scan rates *v* ranging from 0.006 to 0.5 V s^−1^.

The electrochemical behavior of VB_6_ was elucidated based on the CVs recorded for the various scan rates and differential pulse (DP) voltammograms registered for a variety of pHs of the supporting electrolyte. Subsequently, the experimental conditions for VB_6_ determination were thoroughly optimized in order to provide high selectivity, accuracy, and robustness of the developed method. They included the choice of the supporting electrolyte and its pH, as well as the optimization of instrumental parameters for the DP technique. Unless otherwise specified, the DP voltammograms were recorded in the 0.1 mol L^−1^ phosphate buffer (pH 6.6) in the potential range from *E*_0_ = 0.4 V to *E*_e_ = 1.05 V, using the step potential *E*_s_ = 4 mV, pulse amplitude *dE* = 40 mV, waiting time *t*_w_ = 20 ms, and the current sampling time *t*_s_ = 10 ms. In every experiment, the anodic scan was followed by the cathodic one, and the volume of the tested solution was 5 mL.

In the interference studies, the appropriate volume of 1.0 mol L^−1^ standard solution of K^+^, Na^+^, Mg^2+^, Cl^−^, NO_3_^−^, and SO_4_^2−^ ions were added to the supporting electrolyte containing 0.5 mg L^−1^ VB_6_ with the aim to obtain inorganic ions concentration ranging from 2.0 to 200 mmol L^−1^. The influence of organic surface-active compounds was tested by the addition of a proper volume of 10 mg L^−1^ solution of Triton X-100, SDS, and CTAB, resulting in 10-, 50-, 100- and 500-fold excess of a particular compound in comparison to VB_6_ concentration (0.5 mg L^−1^). In the way described above, the possible interference effect of glucose, caffeine, and humic acid, as well as vitamin B_1_, B_3_, B_5_, and B_12_, were established with the use of their standard solution at the concentration of 1.0 g L^−1^ and 10- to 400-fold excess in comparison to VB_6_ concentration. To examine the influence of titanium dioxide, starch, and magnesium stearate on the signal recorded for the solution containing 0.5 mg L^−1^ VB_6_, the appropriate weighted amount (from 1 to 5 mg) of each powder was added directly to the quartz cell. As the tolerance limit for each tested substance, the percentage difference between peak current recorded for VB_6_ without and with interferents less than ±5% was chosen.

For quantitative determination of VB_6_ in pharmaceuticals, 50 μL of the corresponding solution was transferred to 5 mL of the supporting electrolyte. Measurement performed according to the standard addition method included the registration of DP voltammograms for blank and after the introduction of the sample and subsequent additions of the VB_6_ standard solution. For every step, three consecutive scans were recorded, averaged, and subjected to the background correction. Based on the standard addition plot, the concentration of the VB_6_ in the tested solution was calculated as the intercept with the horizontal axis. Taking account of the dilution factor, the content of VB_6_ in a single tablet was computed. In the case of Magne B6 and MagB6forte, the VB_6_ content was verified by UV-Vis measurements [7]. The same voltammetric procedure, with the adjusted volume of the added sample, was applied for VB_6_ determination in the *Oshee* energy drink.

The certified reference materials (CRMs) of surface water and waste water did not contain VB_6_. To test the possibility of VB_6_ determination in these matrices, both CRMs were spiked with VB_6_ standard solution to obtain concentrations of 15 and 0.75 mg L^−1^. Such prepared samples were analyzed in similar manner as described above for pharmaceuticals.

## Results and discussion

### Materials characterization

**Fig. 1:**
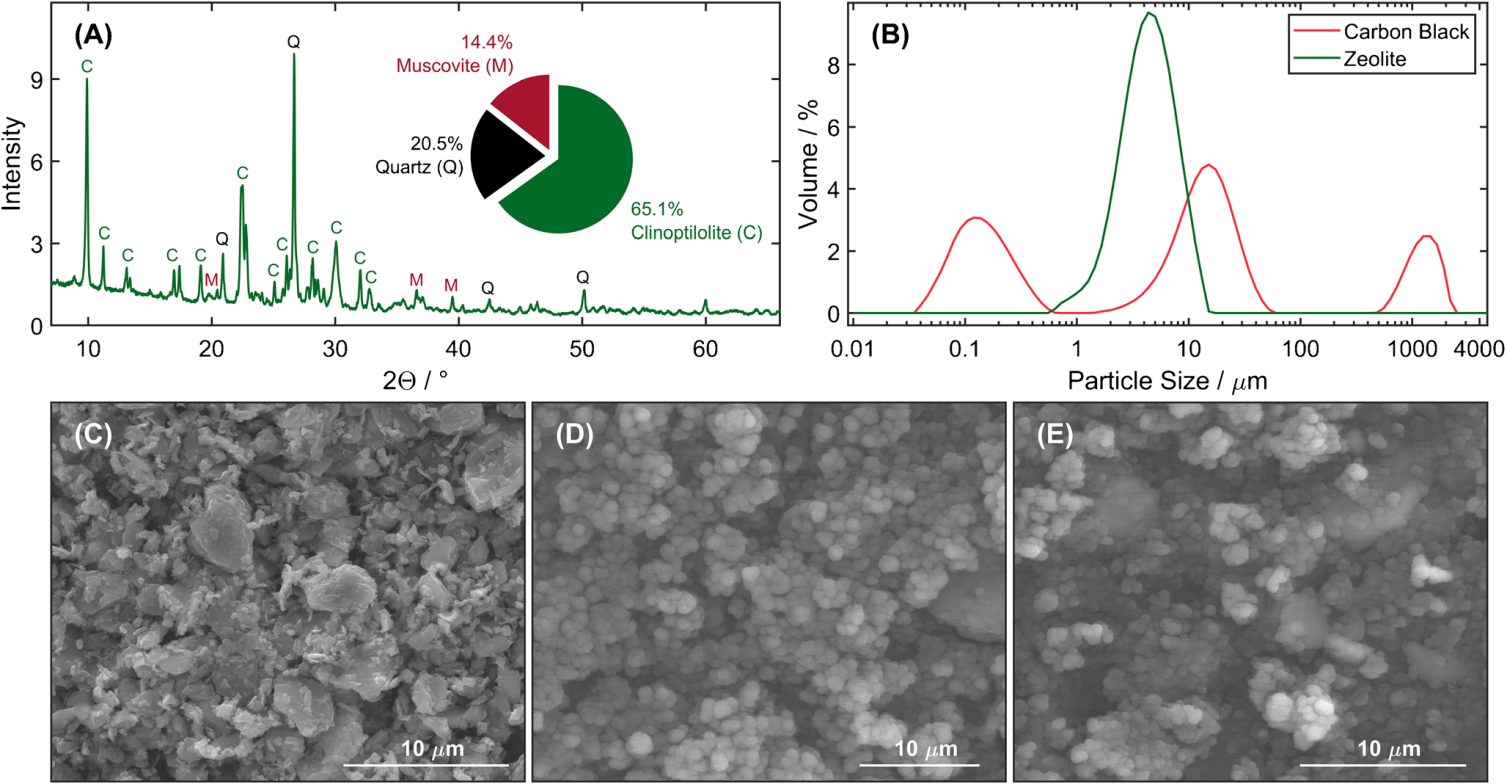
Material characterization. **A** XRD pattern of the used zeolite material. **B** Particle size distribution of the employed carbon black (CB) and zeolite. **C** SEM images of the zeolite, **D** carbon black, and **E** Ni-zeolite/CB modifying layer

The obtained XRD pattern (Fig. 1A) and Rietveld’s quantitative analysis confirmed the dominating content of the Ca-clinoptilolite phase, equal to 65.1%, in the used volcanic-sedimentary origin zeolite material from Sokirnyanskyy deposit. In addition to the zeolite, quartz (20.5%) and muscovite (14.4%) were also determined as impurities.

The particle size distributions (PDSs) of zeolite and CB determined by the DLS method (Fig. 1B) confirm the micro-sized character of their particles. Moreover, the received results show differences in the physical characterization of zeolite and CB particles, where unimodal and bimodal PSD were observed, respectively. As regards the zeolite, the dominant fraction constitutes the particles with a diameter of about 5 μm, whereas for CB particles ranging from 1 to 40 μm with a dominant diameter of about 15 μm can be distinguished.

The specific surface area and porosity of the zeolite and CB, determined from nitrogen adsorption isotherms (Fig. S1 in the Supplementary Materials), are summarized in Table 1. It can be seen that the conductive CB is characterized by the BET surface area and the external surface remarkably higher than zeolite. Moreover, the obtained average pore diameter revealed nanoporous nature of the zeolite and CB, which can be classified as mesoporous materials.

**Table 1:** Textural properties of zeolite material and conductive carbon black

Material	Surface area (m^2^ g^−1^)			Pore volume (cm^3^ g^−1^)		Average pore diameter (nm)
	*S*_BET_	*S*_micro_	*S*_ext_	*V*_micro + mezo_	*V*_mico_	
Zeolite	15.67	3.02	12.6	0.07	0.002	18.82
Carbon black	1133.11	78.57	1054.5	1.79	0.027	6.31

*S*_*BET*_, BET surface area; *S*_*micro*_, micropore area; *S*_*ext*_, external surface area; *V*_*micro+mezo*_, micropore+mezopore volume; *V*_*mezo*_, mesopore volume

The morphology of zeolite and CB particles, as well as Ni-zeolite/carbon black modifying layer on the GCE surface, is presented in Fig. 1. In the SEM image of zeolite (Fig. 1C), a typical lamella structure of clinoptilolite-heulandite family minerals with characteristic flake-shape particles and conglomerates of compact crystals was observed. On the other hand, the CB structure (Fig. 1D) reveals the presence of more or less spherically primary carbon particles, which form three-dimensional, branched aggregates and agglomerates. Regarding Ni-zeolite/carbon black modifying layer (Fig. 1E), the clinoptilolite crystals seem to be covered with CB agglomerates, and together they provide a full coating of the GCE surface. Through the analysis of EDS spectra of zeolite before and after the ion-exchange process (Fig. S1C), nickel and manganese content in zeolite structure amount to 1.12 and 0.76 at. %, respectively, were established.

### Electrodes electrochemical characteristics

The replacement of the Mn^2+^ cations by the Ni^2+^ in the zeolite framework resulted in the extension of the range of available potentials. Moreover, Ni^2+^ ions contribute to a more favorable performance of the modified electrode in terms of its stability and background current. The comparative study with the use of graphite and carbon black indicated that the latter is a better electron-conducting material, since for the model [Fe(CN)_6_]^4−/3−^ redox system it provided the cyclic voltammogram of theoretical shape, with the peak current higher than for the bare GCE. Additionally, the impedance measurements proved that CB lowers the charge-transfer resistance, meaning that the electron transfer can occur more easily. Based on the Randles-Sevcik equation, the surface of the NiZCB-GCE and GCE were established to be 13.4 and 7.1 mm^2^, respectively. For a more detailed investigation of the electrochemical properties of the NiZCB-GCE and the influence of particular components of the modifying layer on the electrode performance, please refer to chapter S2 in the Supplementary Materials.

**Fig. 2:**
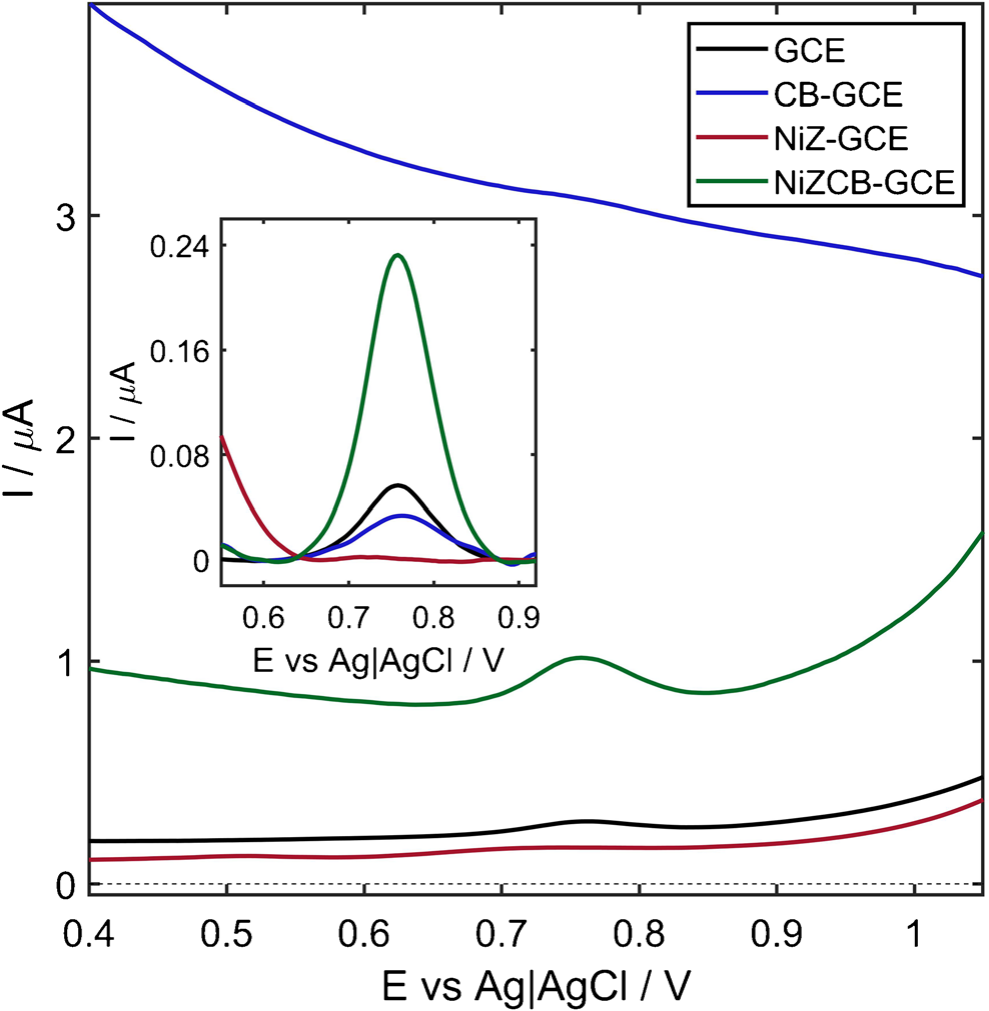
Comparison of DP voltammograms recorded for 0.5 mg L^−1^ VB_6_ (3.0 μmol L^−1^) on the bare GCE, carbon black (CB-GCE), Ni-zeolite (NiZ-GCE), and Ni-zeolite/carbon black modified GCE (NiZCB-GCE). Inset: DP voltammograms subjected to the background correction. Experimental conditions: *E*_0_ = 0.4 V, *E*_e_ = 1.05 V, *E*_s_ = 4 mV, *dE* = 40 mV, *t*_w_ = 20 ms, *t*_s_ = 10 ms. Supporting electrolyte: 0.1 mol L^−1^ phosphate buffer (pH 6.6)

The advantageous performance of the NiZCB-GCE is additionally confirmed by the DP voltammograms recorded for 0.5 mg L^−1^ VB_6_, depicted in Fig. 2. For starters, the signal was registered on the GCE, which acted as the substrate for deposition of the modifying layer. It resulted in a well-developed signal, corresponding to the oxidation of VB_6_, with a relatively low background current. When the surface of GCE was covered with the modifying layer containing only Ni-exchanged zeolite (NiZ-GCE), no analytical signal of VB_6_ was noted. On the other hand, the presence of carbon black on the GCE surface (CB-GCE) resulted in a highly elevated background signal, on which the analytical signal in the form of a small inflection was observed. Finally, the introduction of both the Ni-exchanged nanoporous zeolite/carbon black hybrid nanocomposite into the modifying layer lowered the capacitive current and provided a more stable background signal and higher signal-to-noise ratio. Further improvement is evident in the signals obtained after the background correction, which are presented in the inset in Fig. 2. With respect to the bare GCE, NiZCB-GCE provided ca. 4-times higher response despite having only two-times higher electroactive surface, whereas in the case of the CB-GCE, the signal decreased by ca. 30%. These observations proved that the superior properties of the NiZCB-GCE are evoked by the synergistic effect resulting from the combination of the Ni-exchanged nanoporous zeolite with the conductive CB.

### Mechanism and optimization study

**Fig. 3:**
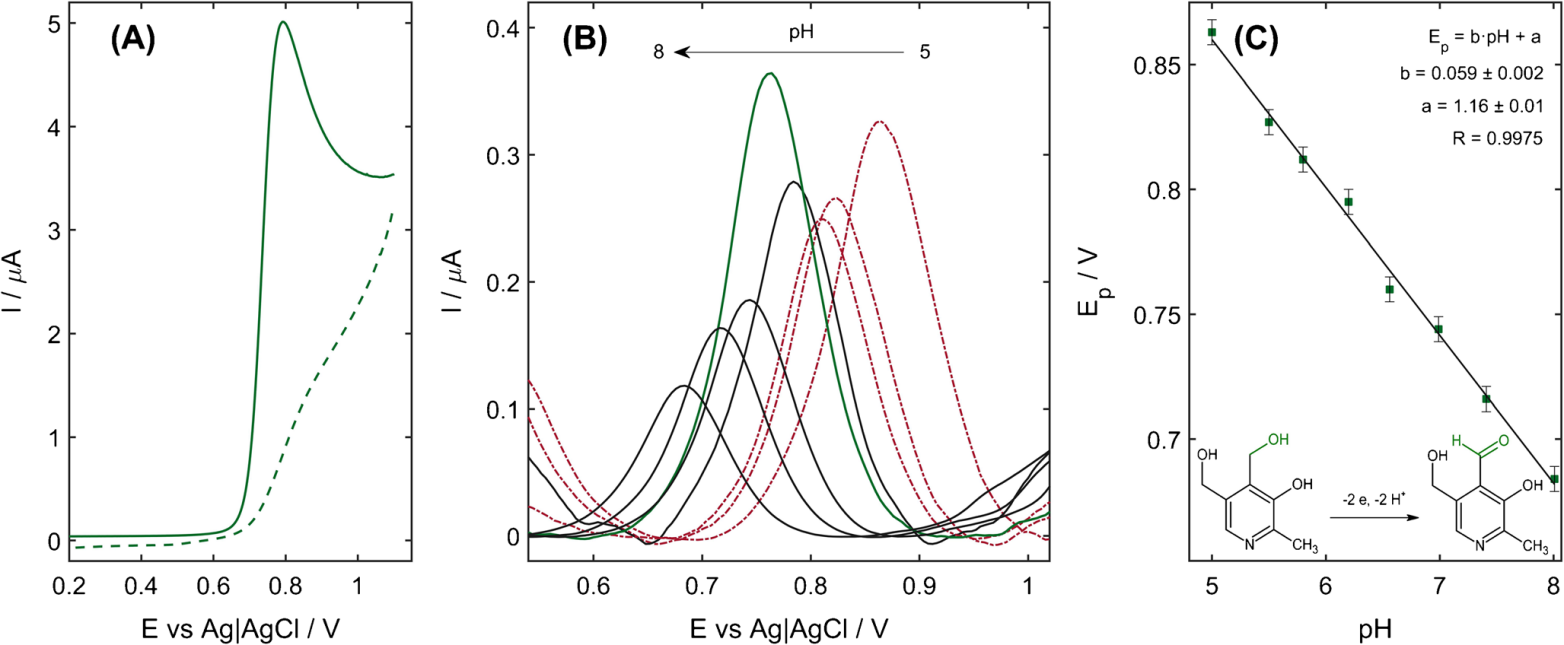
**A** Cyclic voltammogram recorded for 20 μM VB_6_ in 0.1 M phosphate buffer (pH 6.6) using the scan rate of 25 mV s^−1^. **B** The influence of the pH of the acetate buffer (red, dotted curves) and phosphate buffer (black and green solid lines) on the DP signal of 1.0 mg L^−1^ VB_6_ oxidation recorded on the NiZCB-GCE. **C** The relationship between the peak potential and buffer pH value. Inset: the mechanism of VB_6_ oxidation

In the CVs recorded for the VB_6_ in the 0.1 mol L^−1^ phosphate buffer (pH 6.6), only an anodic peak of VB_6_ oxidation was observed (Fig. 3A). The lack of a cathodic peak indicates that the process is chemically irreversible. Moreover, with the increase in scan rate, peak potential shifted towards more positive values, which is a sign of an irreversible oxidation process. In this case, the difference between the peak potential *E*_p_ and the potential *E*_p/2_ at which the currently reached half of the peak current is connected with the number of the exchanged electrons *n* and the charge-transfer coefficient *α* by the equation [36]:

$$ \left|{\mathrm{E}}_{\mathrm{p}}-{\mathrm{E}}_{\mathrm{p}/2}\right|=47.7\mathrm{mv}/\left(\upalpha \mathrm{n}\right) $$

where *α* equals 0.5. For VB_6_, |*E*_p_ − *E*_p/2_| equaled 47.5 mV; thus, the number of the transferred electrons was calculated to be equal to 2.

Furthermore, the influence of pH of the supporting electrolyte was investigated. As illustrated in Fig. 3B, with the increase of pH the peak potential *E*_p_ shifted towards more negative values. The relationship between *E*_p_ and pH was linear (Fig. 3C) with the slope of (0.059 ± 0.002) V per unit change in pH, which is equal to the theoretical value for the electrode processes that involve the equal number of electrons and protons. This leads to the conclusion that during the oxidation of pyridoxine (the studied form of VB_6_), two electrons and protons are detached, resulting in the formation of pyridoxal [21]. The mechanism is depicted in Fig. 3C.

**Fig. 4:**
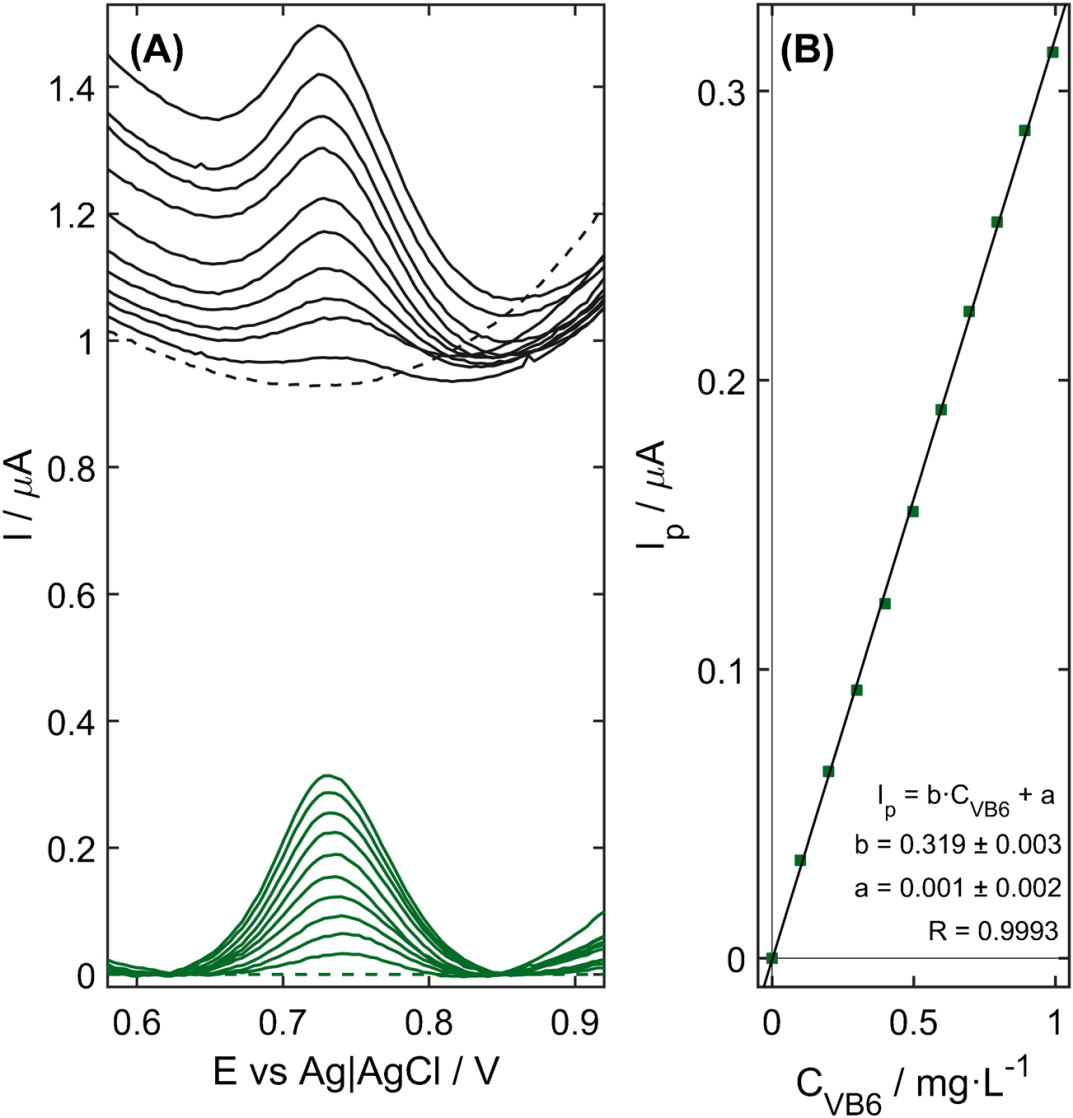
**A** DP voltammograms recorded on the NiZCB-GCE for VB_6_ concentrations ranging from blank to 1.0 mg L^−1^ (black lines) with the curves obtained after the background correction (marked in green). **B** Calibration plot with the regression equation. Experimental conditions as in Fig. 2.

Due to the presence of several functional groups in the VB_6_ molecule, it is involved in the acid-base and hydration equilibria; therefore, the choice of the supporting electrolyte was crucial. For this reason, the pH of tested acetate and phosphate buffers was kept between 5.0 and 8.9, which are two pK-values of VB_6_ [21]. This ensured the same chemical form of VB_6_ in every experiment. As presented in Fig. 4A, the peak current depended on the pH of the supporting electrolyte. Higher values were obtained in the acidic environment with the maximum pH equal to 6.56. Above that, a decrease in peak current with raising pH was observed. Therefore, 0.1 mol L^−1^ phosphate buffer (pH 6.6) was chosen as the best-supporting electrolyte and has been used in further measurements.

To ensure the best sensitivity and reproducibility of VB_6_ determination, univariate optimization of the instrumental parameters was performed for 0.5 mg L^−1^ VB_6_ in 0.1 mol L^−1^ phosphate buffer of pH 6.6. Based on the results presented in section S3 in Supplementary Materials, the most favorable parameters were established to be as follows: the step potential *E*_s_ = 4 mV, pulse amplitude *dE* = 40 mV, waiting time *t*_w_ = 20 ms, current sampling time *t*_s_ = 10 ms, and were applied in the following experiments.

### Analytical performance

With the aim to verify the analytical usefulness of NiZCB-GCE, DP voltammograms for the increasing concentration of VB_6_ from blank to 1.0 mg L^−1^ were registered under the optimized conditions. For the achieved curves (Fig. 4A), the background subtraction step with a 3rd-degree polynomial function was performed, following which calibration graph was plotted. As can be seen in Fig. 4B, the oxidation peak current at the potential of +0.72 V vs. Ag | AgCl | 3 M KCl exhibits a good linear dependence on the concentration of VB_6_ in the range from 0.050 to 1.0 mg L^−1^ (0.30–5.9 μmol L^−1^) with a sensitivity and correlation coefficient (*R*) of 0.319 ± 0.003 μA L mg^−1^ (2.38 ± 0.02 μA L mg^−1^ cm^−2^) and 0.9993, respectively. The limit of detection (LOD) equal to 15 μg L^−1^ (0.09 μmol L^−1^) was calculated according to the following equation: LOD = 3*SD*/b, where *SD* is the standard deviation of current for the blank and *b* is the slope of the calibration curve. The limit of quantification (LOQ) expressed as LOQ = 10*SD/b* was estimated to be 50 μg L^−1^ (0.30 μmol L^−1^).

The achieved LOD value of the developed protocol is more favorable than the results presented in most of the previously reported analytical methods used for VB_6_ determination [6–9]. In comparison to some past reports concerning voltammetric sensors of VB_6_, the NiZCB-GCE demonstrates the LOD value lower than those obtained for electrodes modified with metal and metal oxide nanoparticles (Table 2). On the contrary to the other carbon-based materials used for VB_6_ voltammetric sensors fabrication, the introduction of the conductive carbon black and Ni-zeolite into modifying layer leads to over 76-, 16-, 3-, and 4-times lower limit of detection in comparison to the electrodes chemically modified with SWCNTs and MWCNTs, ERGO, and OMC, respectively. Furthermore, the combination of unique properties of CB and nanoporous zeolite results in obtaining the VB6 sensor characterized by a good measurement sensitivity, accuracy, and stable signal. The stability of the NiZCB-GCE was assessed based on the signal recorded for 0.5 mg L^−1^ VB_6_ in 0.1 mol L^−1^ phosphate buffer (pH 6.6) on ten separate electrode copies. For each of them, the RSD of the peak current calculated for four consecutive voltammograms fell into the range between 2.5 and. 5.4%, proving the outstanding repeatability and short-term stability of each electrode. The RSD of the peak currents obtained at all tested copies equals 22%, thus confirming the good reproducibility of the proposed electrode and its preparation procedure.

**Table 2:** An overview on recently reported nanomaterial-based electrochemical sensors of VB_6_ determination

Electrode	Technique	Linear range (mol L^−1^)	LOD (mol L^−1^)	**Ref.**
Au-NPs/CPE^1^	DPV	1.9–110.8·10^−6^	74·10^−9^	[16]
		110.8–257.0·10^−6^		
Nano-Cu/Au^2^	SWV	0.3–2.7·10^−6^	8.7·10^−8^	[17]
Au-CuO/MWCNTs/GCE^3^	DPV	0.79–18.4·10^−6^	0.15·10^−6^	[18]
ZrO_2_-NPs/IL/CPE^4^	DPV	0.8–550·10^−6^	0.1·10^−6^	[19]
SWCNT-SPE^5^	DPV	3.9–72·10^−6^	6.8·10^−6^	[20]
MWCNT-SPE^6^	DPV	2.0–72·10^−5^	1.5·10^−6^	[20]
ERGO/CCE^7^	DPV	1–70·10^−6^	0.3·10^−6^	[21]
OMC/SPCE^8^	DPV	1–200·10^−6^	0.42·10^−6^	[22]
NiZCB-GCE	DPV	0.3–5.9·10^−6^	9·10^−8^	This work

^1^Gold nanoparticle carbon paste electrode

^2^Copper nanoparticle modified poly-crystalline gold electrode

^3^Gold (core)-copper core-shell multi-walled carbon nanotube glassy carbon electrode

^4^ZrO_2_ nanoparticle/ionic liquid carbon paste electrode

^5^Single-walled carbon nanotube screen-printed carbon electrode

^6^Multi-walled carbon

^7^Electrochemically reduced graphene oxide ceramic carbon ceramic electrode

^8^Ordered mesoporous carbon screen-printed carbon electrode

### Interference studies

Based on the conducted interference studies, no significant changes in the height, shape, and position of VB_6_ oxidation peak current were observed with the presence of inorganic ions in the supporting electrolyte. Even at the highest addition of magnesium ions, which are often present in the large quantities in the dietary supplements next to VB_6_, the signal change did not exceed the ±5% tolerance limit. The same findings were made for the addition of powdery starch, titanium dioxide, and magnesium stearate, which constitute one of the basic excipients of pharmaceuticals. The minor interference effect was observed in the case of glucose and caffeine, for which no more than a 10% decrease of the recorded VB_6_ peak current was noted at their 500-fold excess in comparison to VB_6_ concentration. On the other hand, the introduction of humic acid (HA) into the quartz cell resulted in a considerable diminution of the VB_6_ signal (up to 65% at the highest concentration of HA) without any change in the shape and signal position.

Performed study of possible interference of other B group vitamins occurring in multivitamins dietary supplement indicated that vitamins B_1_, B_3_, and B_5_ did not affect the recorded VB_6_ signal, regardless of their concentration. Only the presence of vitamin B_12_ led to a decrease in the peak height of about 30% at its maximum tested excess. However, even in this condition, the VB_6_ signal is still noticeable, and its shape and position, as well as the background current, remained unchanged.

Among the wide range of tested interferents, the most visible suppressive influence on the VB_6_ oxidation peak current was observed in the case of organic surface-active compounds. For the maximum concentration of non-ionic and cationic surfactants, the recorded VB_6_ peak current decreased to 87% and 53% of its initial value for Triton X-100 and CTAB, respectively. On the other hand, the addition of anionic surfactant caused the increase of VB_6_ peak current in the whole tested excess of SDS in the supporting electrolyte. However, together with the increasing concentration of Triton X-100, SDS, and CTAB, a significant increase in the background current and lower repeatability of the recorded signal were observed. Thus, it can be assumed that the presence of surface-active species may make difficulties in the voltammetric determination of VB_6_.

According to obtained results, it can be concluded that the developed method is characterized by high selectivity, thus devised protocol is suitable for VB_6_ determination in real samples.

### Analysis of the real samples

**Fig. 5:**
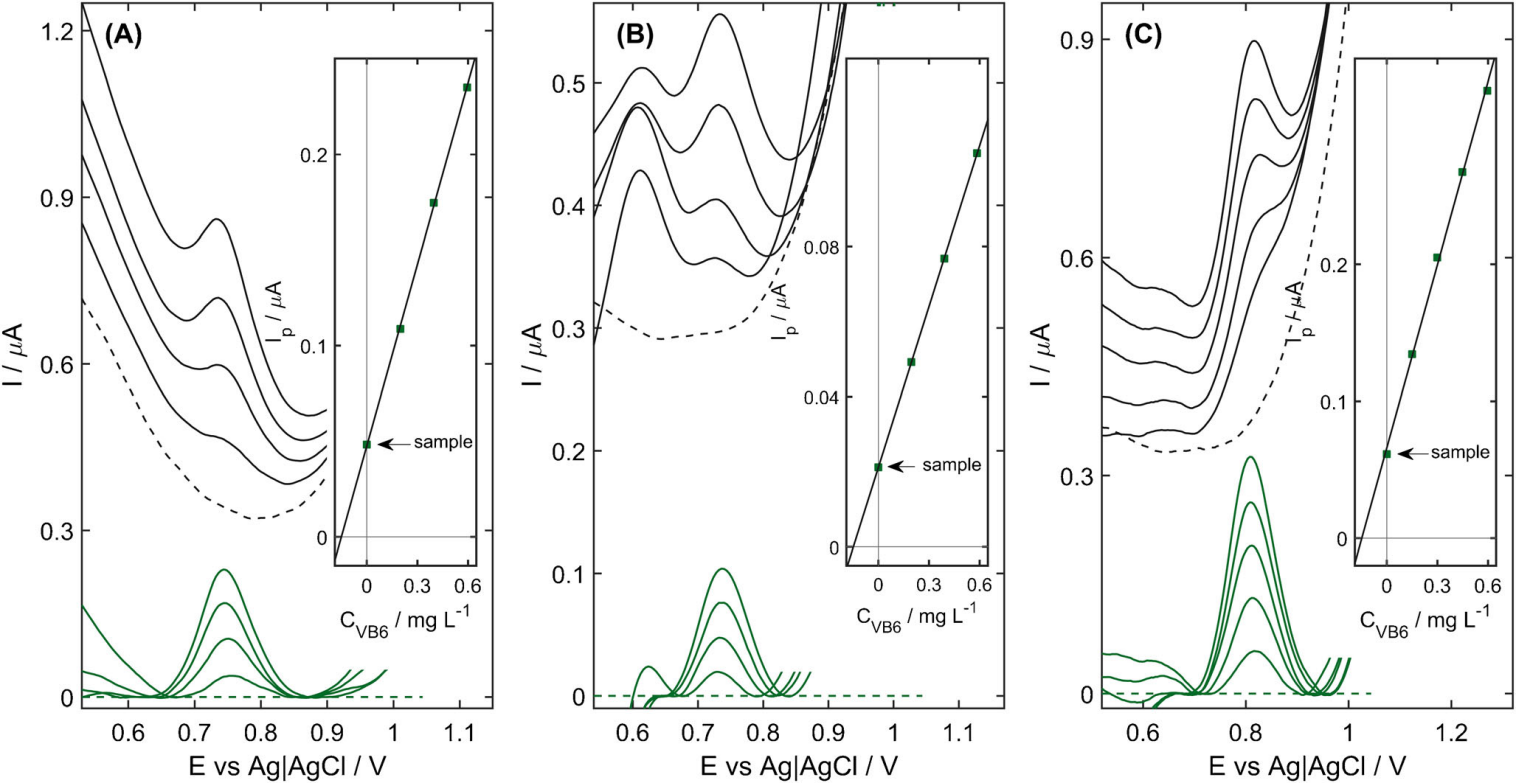
Investigation of the real samples: **A** Vitaminum B Compositum pharmaceutical, **B** *Oshee* energy drink, and **C** wastewater CRM spiked with 0.75 mg L^−1^ VB6. The experimental voltammograms were depicted in black, whereas the curved obtained after the background correction are marked in green. Inset: corresponding calibration plots. Experimental conditions as in Fig. 2

The developed procedure was applied for the determination of VB_6_ in six commercially available multivitamin pharmaceutical formulations. In the potential range, in which the oxidation of VB_6_ proceeds, no other peaks apart from the analytical signal were observed (Fig. 5A). Additionally, the observed current increments after each standard addition prove that the matrix components did not block the surface of the working electrode. The computed contents of VB_6_ in tested pharmaceuticals, summarized in Table 3, are consistent with the amount asserted by the manufacturer. Moreover, in the case of Magne B6 and MagneB6forte, UV-Vis spectroscopy was applied as a reference method, and the obtained values were in accordance with the results of the voltammetric measurements. This proved the accuracy and reliability of the developed protocol of VB_6_ determination. Unfortunately, in the case of the other tested pharmaceuticals, the UV-Vis spectra were dominated by other vitamins, mainly VB_2_ present in a high excess with respect to the VB_6_. Thus, for the sake of selectivity, the fabricated electrode and the reported voltammetric method constitute an excellent alternative for the classical instrumental methods.

**Table 3:** Declared and determined contents of VB_6_ in tested pharmaceuticals with the recovery values

Sample	Declared value of VB_6_ (mg per tablet)	Amount found (mg per tablet)	Recovery (%)
Vitaminum B Complex	1.4	1.32 ± 0.06	94.6
Ballans B Complex	1.4	1.41 ± 0.06	100.6
Bellis B Complex	1.4	1.47 ± 0.02	105.0
MagB6forte	1.4	1.44 ± 0.08	103.1
Vitaminum B Compositum	4.1*	4.15 ± 0.18	100.9
Magne B6	4.1*	4.20 ± 0.03	102.0

*5 mg of pyridoxine hydrochloride

In the case of the *Oshee* energy drink, an additional peak was observed at the potential more negative than the potential of VB_6_ oxidation (Fig. 5B). Nonetheless, thanks to the difference in potentials of these peaks, equal to 0.12 V, the interpretation in the usual manner did not cause any inconvenience. The obtained content equals (0.149 ± 0.004) mg VB6 per 100 mL of the beverage, which is consistent with the declaration of the manufacturer (0.14 mg/100 mL, 106% of recovery).

According to the attached documentation, the certified reference materials (CRMs) of waste and surface water do not contain VB_6_, and no peak was observed in the DP voltammograms in the potential range corresponding to the VB_6_ oxidation. Thus, to verify the capability of NiZCB-GCE to monitor the VB_6_ amount in the environment, the determination of VB_6_ was performed in the spiked CRMs at the level of 15 and 0.75 mg L^−1^ VB_6_. As illustrated in Fig. 5C, a single peak corresponding to VB_6_ oxidation was observed, which in conjunction with the DP voltammogram registered in the non-spiked CRMs proves that none of the components present in the matrix interfered during the determination. The obtained recoveries ranged from 98.7 to 103.1%, and the RSD was below 8% in every examined case. This justified the suitability of the NiZCB-GCE for the quantitative analysis of VB_6_ in surface and waste water samples.

## Conclusion

In this paper, the fabrication and analytical application of a novel voltammetric sensor of vitamin B_6_, based on the GCE modified with Ni-zeolite/carbon black hybrid nanocomposite were described for the first time. The fabricated NiZCB-GCE was obtained by the simple and low-cost drop-casting of Ni-zeolite-carbon black polystyrene suspension onto the surface of the GCE, where no pretreatment of conductive CB is required. The proposed procedure led to the achievement of a durable layer, which offers excellent repeatability and short-term stability, as well as good reproducibility and advantageous electrochemical properties of the developed sensor.

The results of CV and EIS measurements showed that utilization of Ni-exchanged zeolite ensures a wider window of available potentials of the developed sensor in comparison to the application of Mn-form of the zeolite in the modifying layer. At the same time, the replacement of graphite by carbon black results in the more favorable charge-transfer kinetics. Moreover, the decrease of the capacitive current and the amplification of the current response of NiZCB-GCE for VB_6_ proved that superior properties of the proposed sensor are directly connected with the synergistic effect of the nanoporous Ni-exchanged zeolite and conductive CB.

The application of the developed sensor in the selective determination of VB_6_ by differential pulse voltammetry demonstrated the analytical usefulness of NiZCB-GCE, which was confirmed by quantitative analysis of popular supplements samples with very good recovery values. Apart from interference effect of surface-active species, no influence from various inorganic salts (particularly magnesium ions), basic excipients of drugs and pharmaceuticals, and other vitamins of the B group was observed, which makes proposed procedure suitable for the routine analysis or quality control of multivitamin dietary supplements without special sample preparation. The achieved analytical parameters in association with the simple and inexpensive preparation method of NiZCB-GCE make the proposed electrode a highly desirable tool, suitable for usage in the analysis of pharmaceuticals, foods, and environmental samples.

## Supplementary information

ESM 1 (DOCX 399 kb)
