# Supplementary material for: Electrochemical sensor based on Ni-exchanged natural zeolite/carbon black hybrid nanocomposite for determination of vitamin B6
Source: Mikrochim Acta. 2021 Sep 6;188(10):323. doi: 10.1007/s00604-021-04992-x (PMC8420854; doi:10.1007/s00604-021-04992-x)
Supplement: Supplementary file 1 — (DOCX 399 kb) [file 604_2021_4992_MOESM1_ESM.docx]

**SUPPLEMENTARY MATERIALS**

**Electrochemical sensor based on Ni-exchanged natural zeolite/carbon black hybrid nanocomposite for determination of vitamin B_6_**

Radosław Porada, Katarzyna Fendrych^*^, Bogusław Baś

*AGH University of Science and Technology, Faculty of Materials Science and Ceramics, Department of Analytical Chemistry and Biochemistry, Mickiewicza 30, 30-059 Cracow, Poland*

*Corresponding author: fendrych@agh.edu.pl

**S1. Material characterization**


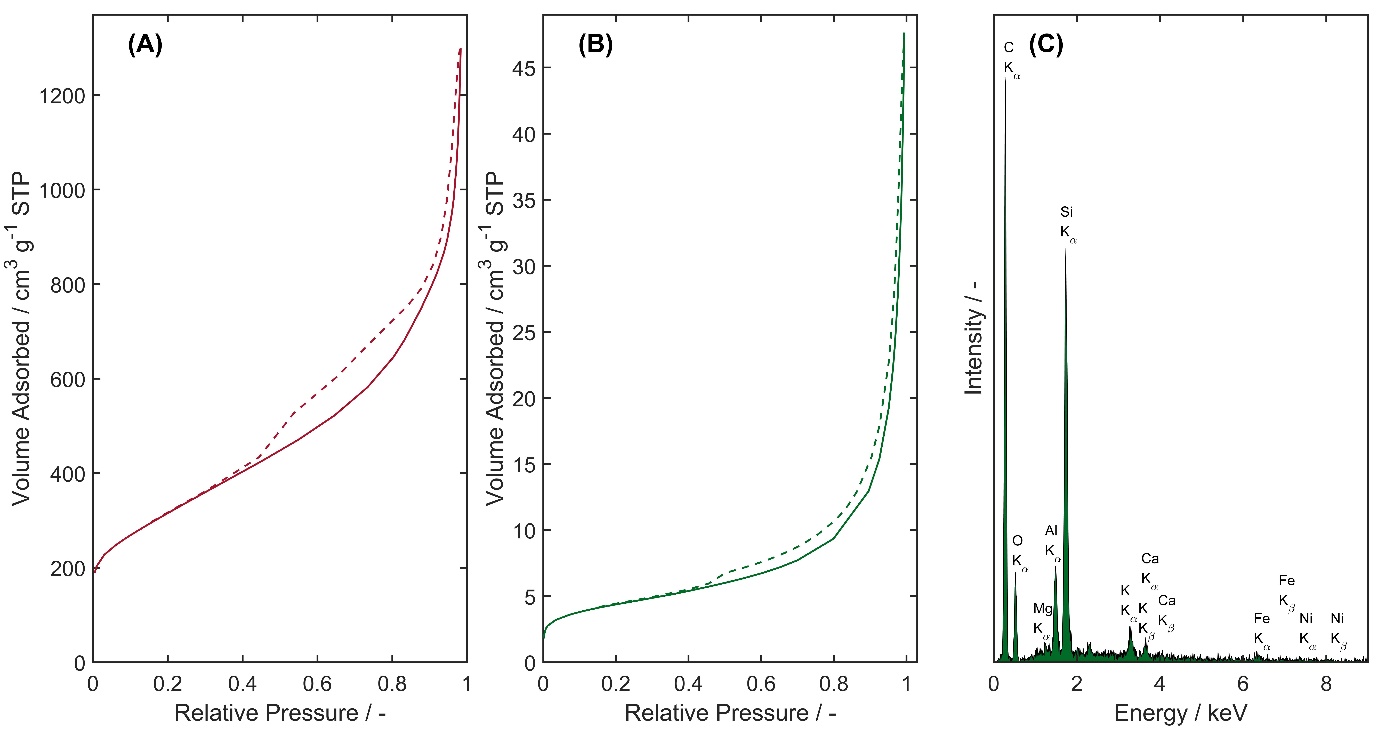


**Fig. S1**.The nitrogen adsorption/desorption isotherms of (A) conductive carbon black and (B) natural zeolite material. Solid lines represent the adsorption, whereas the dashed ones – the desorption. (C) The EDS spectrum of the zeolite after ion-exchange process with Ni^2+^ cations (Ni-zeolite).

The shape of the nitrogen adsorption/desorption isotherms of conductive carbon black and natural zeolite material (Fig. S1A and B, respectively) is most similar to the type IV adsorption isotherm according to the IUPAC classification. This type of isotherm is characterized by the presence of a hysteresis loop, which indicates the phenomenon of capillary condensation taking place in the mesopores of examined materials. The closed hysteresis loop of both CB and zeolite may suggest the presence of cylindrical mesopores with relatively regular shapes [1].

The comparison of the EDS spectrum of the natural zeolite material before (not shown) and after ion-exchange process with Ni^2+^ cations (Fig. S1C) allowed for confirmation of the incorporation of electroactive cations into the zeolite framework structure. In the EDS spectrum of the original zeolite material, peaks connected with tetrahedra atoms (silicon, aluminum, and oxygen) and exchangeable cations (mainly K^+,^ Na^+^, Ca^2+^, and Mg^2+^) were observed. The conducted procedure of zeolite modification resulted in the decrease of the atomic percentage of the elements initially present in zeolite structure with a simultaneous increase of Ni^2+^ content. Based on the atomic concentration of Ni^2+^ at several, different surface points, the average value of nickel content was established. The same procedure was applied for manganese content determination, presented in our previous work [2].

**S2. Electrodes electrochemical characteristics**

The influence of the cation introduced into the zeolite framework on the performance of the fabricated working electrodes is illustrated in Fig. S2A based on the CVs registered in 0.1 mol L^-1^ KCl. In the case of MnZCB-GCE, a peak at +0.3 V, which corresponds to the oxidation of Mn^2+^ cations to Mn_3_O_4_, composing of Mn_2_O_3_ and MnO, and at +0.7 V, resulting from the oxidation of the remaining Mn^2+^ to Mn^3+^, is observed [2]. For the zeolite exchanged with Ni^2+^, no signal was noted apart from the current edges at highly negative and positive potentials, associated with hydrogen and oxygen evolution, respectively. Thus, it is self-evident that the range of the available potentials depends on the cation incorporated into the zeolite framework, and among the tested Mn^2+^ and Ni^2+^ ions, the latter provides more stable functioning in a much wider potential range and ensures a more favorable background current. For these reasons, Ni-exchanged zeolite has been used in further experiments.

In the next step, the effect of the electron-conductive material has been examined. The ideal cyclic voltammogram recorded for the 0.2 mmol L^-1^ [Fe(CN)_6_]^4-/3-^ in 0.1 mol L^-1^ KCl on the GCE became distorted upon deposition of the modifying layer containing zeolite and graphite (Fig. S2B). The separation between the anodic and cathodic peaks, equal to 500 mV, indicates a significant hindrance of the electron transfer across the electrode–electrolyte phase boundary. However, when graphite had been substituted by the carbon black, the cyclic voltammogram regained its theoretical shape with equal cathodic and anodic currents. Their values increased in comparison to the bare GCE, which can be ascribed to a higher surface area and more facile electron transfer of the NiZCB-GCE. The latter is confirmed by the impedance spectra (Fig. S2C), which indicates that the charge-transfer resistance (*R_ct_*) for NiZCB-GCE is ca. 2 times lower than for the bare GCE. The straight line observed in the spectra of GCE and NiZCB-GCE in the lower frequency range corresponds to the diffusion of the analyte to the electrode surface. This segment did not appear in the spectra of NiZG-GCE, where only a huge semi-circle representing the *R_ct_* was noted. This proved the blocking character of the modifying layer containing graphite. The superiority of conductive CB with respect to graphite can be associated with a low wettability of submicrometric fraction of CB by the used suspension (polystyrene with tetrahydrofuran and dichloromethane) and the formation of CB conglomerate, whereby the charge transfer may occur more easily. Thus, CB has been employed in subsequent measurements. The formation of CB conglomerates is proved by the CV recorded on the GCE covered with the modifying layer containing only CB, as its shape resembles the cyclic voltammogram characteristic for spherical diffusion to microelectrodes (Fig. S2B). Nonetheless, this resulted in the slower charge-transfer kinetics, as proved by the corresponding Nyquist plot (Fig. S2C). Thus, it can be concluded that the addition of zeolite is crucial for the homogeneous distribution of CB on the whole electrode surface, so that the electrode can function as a typical macroelectrode.


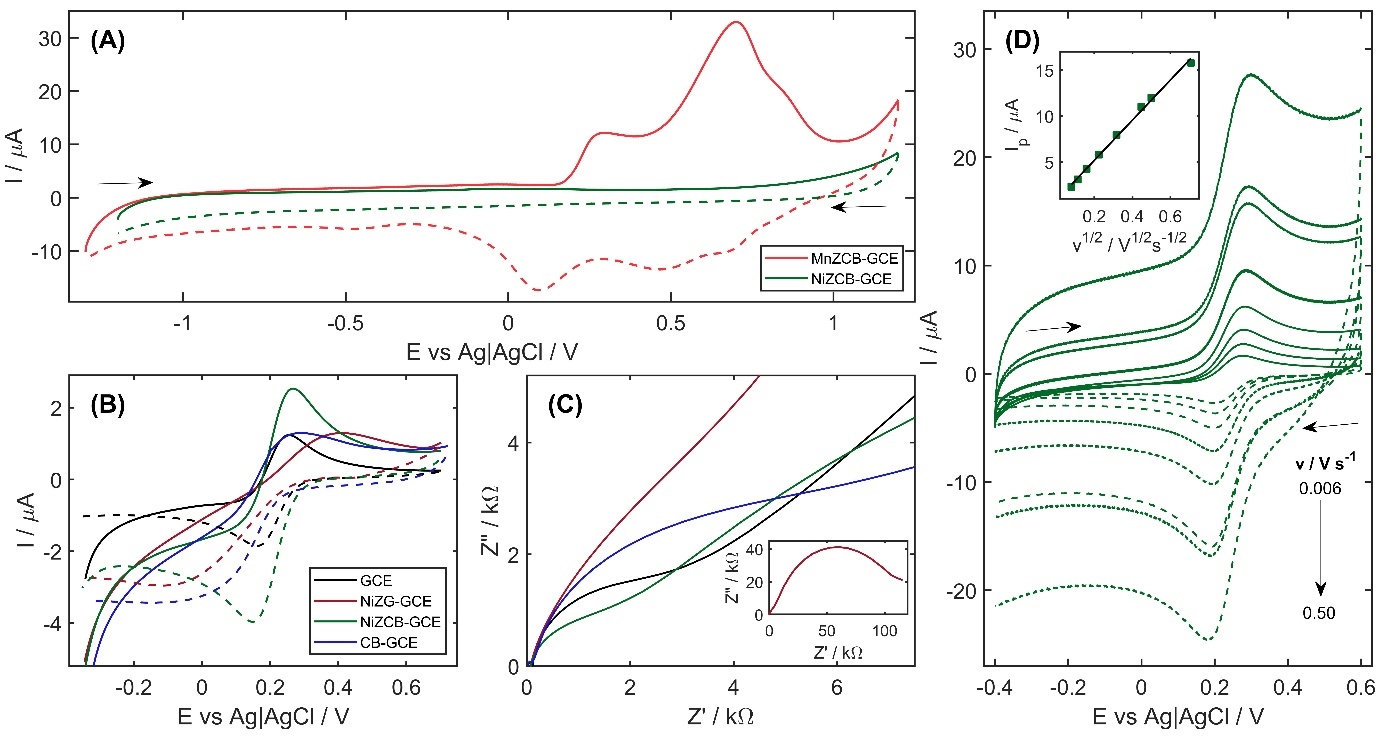


**Fig. S2.** Investigation of the electrochemical properties of the tested electrodes. (A) Cyclic voltammograms (CVs) recorded for Mn-zeolite/carbon black GCE (MnZCB-GCE) and Ni-zeolite/carbon black GCE (NiZCB-GCE) in 0.1 mol L^-1^ KCl (scan rate *v* = 0.025 V s^-1^). Solid lines represent anodic scans, whereas the dashed ones correspond to the cathodic scans. (B) CVs recorded for 0.2 mmol L^-1^ [Fe(CN)_6_]^4-/3-^ in 0.1 mol L^-1^ KCl for bare GCE, Ni-zeolite/graphite GCE (NiZG-GCE), NiZCB-GCE, and carbon black GCE (CB-GCE) (*v* = 0.010 V s^-1^) with (C) corresponding impedance spectra. (D) CVs recorded on NiZCB-GCE for various scan rates (*v*) in 0.1 mol L^-1^ KCl with 0.2 mmol L^-1^ [Fe(CN)_6_]^4-^ redox indicator.

Fig. S2D presents the CVs registered for the NiZCB-GCE in the 0.1 mol L^-1^ solution of KCl containing 0.2 mmol L^-1^ [Fe(CN)_6_]^4-^, using the scan rates *v* from 0.006 to 0.5 V s^-1^. With the increase in *v*, higher peak currents were recorded, and the potential of anodic and cathodic peaks remained unchanged. These features evidenced that the process is reversible and controlled by diffusion. The relationship between the peak current *I_p_* and the square root of *v* was linear. Using the Randles-Sevcik equation, the electroactive surface of NiZCB-GCE was calculated to be equal to 13.4 mm^2^, which is 1.9-times higher than the surface of the GCE (7.1 mm^2^).

**S3. Optimization study**

To ensure the best sensitivity and reproducibility of VB_6_ determination, univariate optimization of the instrumental parameters was performed for 0.5 mg L^-1^ VB_6_ in 0.1 mol L^-1^ phosphate buffer (pH 6.6). For the step potential *E_s_*, tested in the range from 1 to 7 mV, no significant changes in the background current were observed. The peak height increased *quasi*-linearly with the increase in *E_s_* up to 5 mV, afterward a negligible increment was noted. Since the best repeatability was observed for *E_s_* = 4 mV, this value has been applied in all subsequent measurements. The influence of the pulse amplitude *dE* was tested in the range from 10 to 80 mV for both positive and negative directions. In the process, the background level was found to increase with the increase in *dE*, which is associated with the capacitive current being proportional to *dE*. Similarly, higher values of the peak current were recorded for higher amplitudes, and the magnitudes were comparable for the corresponding negative and positive values of the *dE*. Due to all the above-mentioned reasons, *dE* = 40 mV has been selected for further experiments. Finally, the effect of the waiting time *t_w_* and current sampling time *t_s_* in the range from 5 to 40 ms was investigated. It was found that by extending the *t_w_* from 5 to 20 ms, the background current decreased 4-times, whereas the peak height fell down only by 10%. For higher *t_w_* values, no improvement was observed. The sampling time did not significantly influence the peak current (relative change <9%), however, it caused a decrease in the background current. The best repeatability was observed for 10 ms sampling time, therefore, *t_w_* = 20 ms and *t_s_* = 10 ms have been applied in the following measurements.

**Reference:**

1. Alothman ZA (2012) A review: Fundamental aspects of silicate mesoporous materials. Materials (Basel) 5:2874–2902. https://doi.org/10.3390/ma5122874

2. Porada R, Fendrych K, Baś B (2021) Development of novel Mn-zeolite / graphite modified Screen-printed Carbon Electrode for ultrasensitive and selective determination of folic acid. Meas J Int Meas Confed 179:109450. https://doi.org/10.1016/j.measurement.2021.109450
